# The clinical and physiological profiles of kissing stents in endovascular reconstruction of aortoiliac occlusive disease

**DOI:** 10.1113/EP092912

**Published:** 2025-10-29

**Authors:** Christopher J. Goulden, Matti Jubouri, Eka Narayan, Sakshi Roy, Fatima Kayali, Wael I. Awad, Damian M. Bailey, Ian M. Williams, Mohamad Bashir

**Affiliations:** ^1^ Liverpool University Hospitals NHS Foundation Trust Liverpool UK; ^2^ Hull York Medical School University of York York UK; ^3^ School of Medicine Queen's University Belfast Belfast UK; ^4^ Department of Cardiothoracic Surgery, Barts Heart Centre St Bartholomew's Hospital London UK; ^5^ Neurovascular Research Laboratory, Faculty of Life Sciences and Education University of South Wales Pontypridd UK; ^6^ Department of Vascular Surgery University Hospital of Wales Cardiff UK

**Keywords:** aortoiliac occlusive disease (AIOD), aortoiliac reconstruction, bare‐metal stents (BMS), covered stents (CS), endovascular reconstruction, kissing stents

## Abstract

Aortoiliac occlusive disease affecting the abdominal aorta and iliac arteries is conventionally treated with open surgical repair and is the mainstay of treatment. Endovascular techniques have become a less invasive alternative, especially for high‐risk patients. Kissing stents are particularly useful in this situation and involve the placement of two stents at the aortic bifurcation to achieve satisfactory perfusion to both the iliac arteries. These stents can be bare‐metal or covered stents with varied physiological characteristics and differing clinical outcomes. Bare‐metal stents are more prone to restenosis due to neointimal hyperplasia, whereas covered stents minimise tissue ingrowth and are associated with improved patency. Both in vitro and in vivo studies have demonstrated that stent design and deployment significantly influence blood flow patterns, shear stress and long‐term patency. Radial mismatch and turbulent flow have also been reported to impact the durability of kissing stents. Covered stents tend to outperform bare‐metal stents in complex lesions with lower reintervention rates and improved perfusion as indicated by improvement in ankle‐brachial indices. While short‐term outcomes for kissing stents are favourable, especially in high‐risk patients, long‐term patency remains a concern and requires further evaluation. This review evaluates kissing stents’ clinical performance and physiological implications in aortoiliac occlusive disease and discusses anatomical and pathological considerations in selecting the optimal endovascular strategy.

## Abstract (highlights)

- **What is the topic of this review?**
  This review focuses on the endovascular treatment of aortoiliac occlusive disease, specifically examining the use of kissing stents and the covered endovascular reconstruction of the aortic bifurcation technique.
- **What advances does it highlight?**
  The review highlights advances in stent design, particularly the shift from bare‐metal to covered stents and their physiological and clinical implications. The review also highlights how these innovations improve flow dynamics, reduce restenosis, and enhance long‐term patency. Evidence comparing outcomes has been critically evaluated, illustrating how newer techniques like covered stents offer anatomical and haemodynamic advantages over traditional stents in managing complex aortic bifurcation lesions.

## 1 INTRODUCTION

Aortoiliac occlusive disease (AIOD) is a peripheral artery disease which affects the abdominal aorta and iliac arteries (Kimyaghalam et al., 2024). This obstructs blood flow to the lower limbs through stenosed arteries due to atherosclerotic disease. Plaque formation increases the risk of plaque dislodgment, increasing the likelihood of emboli formation. The clinical presentation for AIOD varies from asymptomatic to critical, limb‐threatening ischaemia, with the severity depending on the degree and location of the arterial blockage (Clair et al., 2015).

The treatment of AIOD encompasses a range of surgical and endovascular techniques. These include open surgery, direct aortoiliac reconstruction and extra‐anatomic bypass. Endovascular options include balloon angioplasty with or without stenting on the morphology of the arterial tree. Historically, the traditional treatment for AIOD, particularly when involving aortic bifurcation, has been open surgery. However, the associated risks, including significant morbidity and mortality, often limit its applicability, especially for patients with advanced age, frailty and co‐morbid conditions (Clair et al., 2015). Over the past decade, there has been considerable advancement in endovascular techniques. The endovascular approach has become the first‐line alternative to open surgery, particularly for the treatment of AIOD, as open surgery has become reserved for those where a catheter‐based approach has failed, or the surgical anatomy is challenging. For example, an endovascular approach is not preferred for a short aneurysmal neck (<1.0 cm), narrow aortic angle (<120°), or a diseased aortic neck with calcification or thrombus covering more than 50% of its circumference (Kim et al., 2019).

**FIGURE 1:**
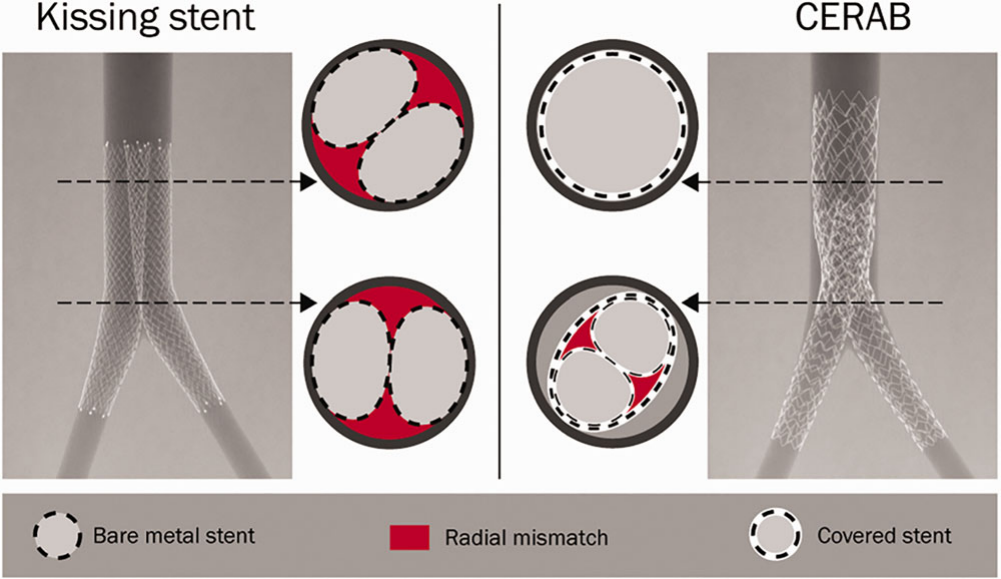
Overview of the KS technique (left) using two self‐expandable stents and the CERAB technique (right) using three balloon expandable stents. For each configuration two cross sections are shown to depict the size of the in vitro radial mismatch. Re‐used from Groot Jebbink et al., 2017. Published under Open Access agreement with Creative Commons Attribution‐NonCommercial 4.0 License.

There are various endovascular techniques for managing AIOD, which include the Covered Endovascular Reconstruction of the Aortic Bifurcation (CERAB) procedure and the use of bilateral ‘kissing’ stents (KSs) with either bare metal stents (BMSs) or covered stents (CSs) (Groot Jebink et al., 2015). These have become increasingly favoured due to the ability to successfully deliver satisfactory long‐term results, such as higher overall primary patency rates at 2 years (96.7% vs. 80.0%) and lower rates of early perioperative complications in the endovascular group (40.0% vs. 6.7%) (Mayor et al., 2019; Yang et al., 2021). However, there was a higher re‐intervention rate when compared to open repair (3.3% vs. 20.0%) (Mayor et al., 2019). While Mayor et al. (2019) report higher primary patency rates with endovascular approaches, the study's non‐randomized design and potential selection bias – favouring higher‐risk patients for endovascular repair – should be considered when interpreting these results (Figure 1).

KSs are deployed at the aortic bifurcation and extend distally into the common iliac arteries. They help reduce the duration of hospital stay and lower 30‐day mortality and morbidity rates and comparable short‐term outcomes (Björses et al., 2008). However, there are concerns, and areas of incomplete contact between the stent and vessel lumen can occur, resulting in the formation of ‘radial mismatch’ or ‘protrusion mismatch’ zones. This phenomenon can lead to reduced durability of the deployed stent (Miklos Vertes et al., 2018; Greiner et al., 2005). The CERAB technique uses three covered stents to preserve the aortic bifurcation and reduces radial mismatch and stent protrusion while providing improved outcomes when compared to KSs (Sharaffudin MJ et al., 2008; Garcia et al., 2021; Groot Jebink, Holewijn et al., 2017; Groot Jebink, Mathai, et al., 2017) (Figure 1).

This review aims to explore in vitro and in vivo evidence of physiological changes to blood flow after different techniques of endovascular reconstruction of the aortic bifurcation, focusing on KSs and how these may impact clinical outcomes. To achieve this, a comprehensive literature search was conducted across PubMed, Scopus, Embase and Ovid databases from inception to March 2025. Keywords included ‘Kissing Stents’, ‘Aortoiliac Occlusive Disease’, ‘Covered Stents’, ‘Bare‐Metal Stents’ and ‘Endovascular Reconstruction’. Inclusion criteria were peer‐reviewed studies, systematic reviews, meta‐analyses, randomised controlled trials, in vitro studies, and relevant observational studies focused on KS or CERAB techniques in AIOD. Studies not published in English, case reports and non‐peer‐reviewed materials were excluded. Reference lists of key studies were also screened to capture additional relevant literature. It is important to note that many existing studies were found to suffer from limited sample sizes, short follow‐up periods, and lack of blinding or randomisation, introducing bias risks.

## 2 KISSING STENT‐GRAFTS IN AOID: A CLINICAL OVERVIEW

Endovascular treatment at the aortic bifurcation is a cornerstone in managing AIOD, with KSs being a critical technique for durability. The technique has its origins in the need to maintain the patency of bifurcated arterial pathways where the abdominal aorta divides into the common iliac arteries – a site commonly affected by atherosclerotic disease (Serefli et al., 2021; Sharafuddin et al., 2008). The expression ‘kissing stents’ has been derived from the visual interlocking configuration of two stents at the bifurcation point. Hence, the term ‘kissing stents’ aptly describes their deployment and where they abut or ‘kiss’ in the middle (Moon et al., 2015; Serefli et al., 2021). This restores the natural anatomy, preserves the essential blood flow distally and provides a scaffold keeping the vessel open and ensuring normal blood flow into each leg (Ahmad et al., 2022; Moon et al., 2015). This section analyses the current body of evidence concerning KSs and how the physiological implications of the process might affect clinical outcomes. Observational studies, clinical trials and retrospective reviews have been analysed to provide an evidence‐based overview of the evolution of KSs. The clinical application and short‐ and long‐term outcomes associated with their use in treating AIOD were also studied.

The immediate outcomes after stenting either with BMSs or CSs relate to technical success and procedural complications. Procedural complications include vessel dissection, stent migration and embolization. Early complications include groin haematoma, infection and formation of pseudoaneurysm. In the mid and long‐term, the comparison includes the return of intermittent claudication, stent occlusion, need for re‐intervention, endovascular or surgical interventions, and failure to salvage the limb measured by the amputation rates (Ahmad et al., 2022). Other outcome measures include primary patency, where no additional procedures are required; assisted patency, where an additional procedure is needed to achieve patency; and secondary patency, where re‐intervention is required to maintain the patency (Nanto et al., 2019). Studies have reported a high technical success rate of 99% in endovascular procedures, with procedural mortality of 2% and 30‐day mortality of 3% (Ahmad et al., 2022). In another study, perioperative complications occurred in 9.8% of cases, including two deaths, renal issues, embolization and access site complications. The long‐term outcomes showed promising results, with primary patency rates of 88%, 70% and 70% at 1, 3 and 5 years, respectively, while secondary patency rates were higher at 98%, 87% and 77% (Nanto et al., 2019).

The advantages of KSs have explicitly been highlighted in patients with limited life expectancy and high surgical risk. For example, hospitalisation duration is significantly shorter compared to surgical revascularisation, almost by a mean of 7 days (Lee et al., 2023). The KS technique is also associated with low morbidity and mortality rates and can be considered an alternative to open repair (Björses et al., 2008). Restenosis, however, remains a concern with KSs. A study has identified that a stent diameter >7 mm is a significant protective factor against restenosis. Further analysis showed that severe calcification is the sole significant determinant of restenosis, underscoring its critical role in patient outcomes (Shen et al., 2023).

While procedural advantages are present, there are concerns regarding the long‐term outcomes and durability of KSs, particularly in comparison to the established benefits of open surgery. Unfavourable outcomes following iliac artery stenting are rare but require attention. A study reported ischaemic events in asymptomatic limbs in 6% of cases, with late stent thrombosis (>30 days) and downstream infra inguinal disease progression accounting for 3% each (Ahmad et al., 2022). Patency rates in the short term are acceptable; however, there is a need for further research to address factors contributing to occlusion and durability of the intervention, as the long‐term patency rates for KSs decline over time, with primary patency reported at 89.3% at 12 months, 78.6% at 24 months and 69.0% at 60 months (Groot Jebbink, Holewijn et al., 2017).

Despite limitations in medium‐ and long‐term patency, the KS technique is successful in high‐risk patients with limited life expectancy as it has a shorter recovery time and reduced operative stress (Greiner et al., 2003). Importantly, no long‐term occlusions in previously asymptomatic or non‐diseased limbs were reported in patients treated with kissing stents. Primary assisted and secondary patency rates in diseased limbs were 96%, 84%, and 84%, respectively. In non‐diseased limbs, rates were 92% and 100%, demonstrating the technique's ability to maintain patency in robust non‐diseased vascular territories (Mohamed et al., 2002). Although reinterventions were occasionally required, they did not detract from the favourable outcomes in non‐diseased limbs (Mohamed et al., 2002).

### 2.1 The types of kissing stents

The classification of KSs falls into two categories: BMSs and CSs, each with distinct characteristics, applications and implications for patient outcomes. Both have been manufactured in various forms to address the complex anatomy and flow dynamics of the diseased aortic bifurcation. The choice between BMSs and CSs in KS procedures affects the immediate and long‐term outcomes.

BMSs are composed of a metallic mesh without additional covering and are favoured for flexibility. This allows them to conform to the contours of the blood vessel and has proven effective in restoring blood flow. However, concerns have been raised regarding their long‐term efficacy, as they can be compromised by neointimal hyperplasia (Kobo et al., 2020).

This can lead to restenosis, a phenomenon well‐documented in multiple studies (Moon et al., 2015; Serefli et al., 2021). Furthermore, in the specific context of KSs, BMSs may present challenges related to stent apposition and the potential for creating areas of turbulent flow at any stent overlap region predisposing to thrombotic events (Ahmad et al., 2022; Hinnen et al., 2015).

CSs are manufactured with metallic scaffolding enveloped by a synthetic fabric or graft material. This design aims to reduce the incidence of restenosis by preventing tissue in‐growth through the stent struts. According to studies, restenosis rates associated with BMSs range from 17% to 41%, making it a common occurrence (Buccheri et al., 2016). Reassuringly, CSs have long‐term patency comparable with surgical bypass, possibly because they provide a physical barrier against neointimal proliferation. These characteristics may improve long‐term patency and reduce the need for repeat interventions (Boulitrop et al., 2020; Torrealba et al., 2023). The use of CSs when KSs are deployed has shown promising results regarding both patency and low postoperative complications (Torrealba et al., 2023).

### 2.2 Comparison of bare‐metal and covered stents

The comparative efficacy of CSs versus BMSs in aortoiliac occlusive disease has been assessed in several key studies, including the landmark covered versus balloon expandable stent trial (COBEST). This randomized controlled study evaluated outcome based on the TransAtlantic Inter‐Society Consensus (TASC) II classification, categorising aorto‐iliac and femoro‐popliteal lesions into four groups (A–D). The TASC II classification categorizes arterial lesions by severity and location, guiding treatment decisions. Type A and B lesions are less complex, with excellent to good outcomes using endovascular or endoluminal approaches. In contrast, type C and D lesions are more extensive or calcified, often requiring surgical management for better long‐term results, with endovascular techniques reserved for high‐risk surgical patients. The COBEST trial demonstrated comparable outcomes between CS and BMS TASC B lesions but superior long‐term patency (hazard ratio (HR), 8.639; 95% CI, 54.253–75.753; *P* = 0.003) and clinical outcomes such as reduced restenosis (HR, 0.136; 95% CI, 0.042–0.442) with CSs for TASC C and D lesions. The 5‐year follow‐up data confirmed this sustained advantage of CSs, showing reduced revascularization rates (odds ratio (OR), 21; 95% CI, 0.07–0.64; *P* = 0.006) (Hardman et al., 2014; Mwipatayi et al., 2011; Mwipatayi et al., 2016). However, limitations of the COBEST trial include its single‐centre design, relatively small patient cohort (125 patients) and the evolving nature of stent technology during the study period (2004–2008), which may limit generalisability to current practice. Moreover, lesion morphology heterogeneity and operator experience were not uniformly controlled, introducing potential confounders affecting outcomes.

Data from the ILIACS Registry examining KS configurations at the aortic bifurcation revealed that while 3‐year patency rates were similar between CSs and BMSs, CSs showed particular advantages in complex lesions with moderate to severe calcification and provided better protection against iliac rupture (0% vs. 3.5%; *P* = 0.013), with greater improvements in ankle‐brachial pressure index (ABPI) (0.43 ± 0.22 vs. 0.36 ± 0.24; *P* = 0.02) (Squizzato et al., 2021). As a multicentre observational registry, the ILIACS data provide valuable real‐world insight, yet are subject to inherent selection bias, varying operator techniques and lack of randomization. Additionally, confounding factors such as differing stent types, lesion calcification severity and patient comorbidities may influence outcomes, complicating direct comparisons between CS and BMS groups. Meta‐analysis on the subject has revealed that in aortoiliac disease, CSs yielded significantly improved ABPIs (mean deviation (MD), 0.08; 95% CI, 0.07–0.09; *P *< 0.001) and lower reintervention requirements versus BMSs (OR, 0.19; 95% CI, 0.09–0.42; *P *< 0.001), with no significant differences observed in technical success, complication rates, limb salvage and patient survival (Hajibandeh et al., 2016). The meta‐analysis synthesized heterogeneous studies with variable definitions of primary endpoints, inconsistent follow‐up durations and different stent platforms. These factors limit the strength of pooled estimates. Additionally, publication bias cannot be excluded, as smaller negative studies are often under‐reported. A more recent meta‐analysis that included 10 studies with a total of 1695 limbs further confirmed that target lesion revascularization was significantly reduced with CSs. However, technical success, limb salvage, overall survival as well as primary and secondary patency rates were largely similar and a statistical difference between the two groups was not found (pooled risk difference (RD), 0.00; 95% CI, −0.01 to 0.01; *P* = 0.710) (Zeng et al., 2023). Although this updated meta‐analysis included 10 studies encompassing 1695 limbs, heterogeneity remained a concern, particularly regarding lesion complexity, operator skill levels, and stent types. Notably, the authors reported no significant difference in several endpoints, possibly reflecting selection bias or underpowered subgroup analyses. Interpretation should therefore be cautious, especially for generalization across all AIOD patients.

The decision between BMSs and CSs for KS procedures must be considered according to the patient's pathology and vascular anatomy. Also, a clear understanding of each stent type's benefits and potential risks must be understood.

### 2.3 Innovations in the design of kissing stents

There have been recent advancements in KS design to improve on the perceived limitations of earlier stents. Newer stents have been developed to provide better anatomical fit and reduce any radial mismatch, which can have deleterious effects on blood flow (Groot Jebbink et al., 2015). For example, stents with improved flexibility and radial force may offer a more physiological reconstruction and conform more closely to the aortic bifurcation's natural geometry (Groot Jebbink, Ter Mors, et al., 2017). The available literature concerning KS is extensive and has provided significant clinical data, technical details and outcome analysis.

## 3 THE IMPACT OF KISSING STENTS ON THE VASCULAR PHYSIOLOGY

The procedural success of KSs owes much to their ability to optimise haemodynamics and minimise complications such as restenosis. The interaction between the stents’ structure, the arterial wall and the blood flow dynamics are integral to optimum outcomes. The stent radial force, malposition and geometric alignment all contribute to the complex physiological changes post‐intervention (Groot Jebbink et al., 2017).

Using KSs in the endovascular treatment of AIOD requires a deep understanding of their impact on vascular physiology. Comparative studies have been a prerequisite to understanding the influence of BMS and CS KSs on blood flow, and these have concluded that the complexity of the lesion determines the outcome and dictates the most appropriate stent. In vitro simulations employing computational fluid dynamics (CFD) compared the flow profiles between different stent types, which has been instrumental in understanding the haemodynamic effects of different stent configurations, providing a controlled environment to assess flow patterns, wall shear stress and any impact on blood flow (Groot Jebbink, Ter Mors, et al., 2017). This highlighted the potential advantages of CSs with natural flow patterns demonstrated. These findings have been repeated in vivo, providing insights into patient outcomes, which studies the patient anatomy, disease severity and durability of stent performance (Soga et al., 2020; Torrealba et al., 2023). These studies demonstrated the clinical benefits of CSs, including lower intervention‐related complication rates and superior long‐term vessel patency (Abdelbaqy et al., 2022; Groot Jebbink, Ter Mors, et al., 2017). This section explores the in vitro and in vivo evidence of physiological changes to blood flow following the deployment of different types of KS.

In vitro studies have principally scrutinised the immediate effects on flow restoration. Whilst BMSs can treat arterial occlusions, they can cause significant changes in flow patterns, particularly at the site of any stent overlap. These alterations can lead to increased shear stress on the vessel wall, potentially accelerating neointimal hyperplasia and subsequent restenosis (Groot Jebbink et al., 2015; Moon et al., 2015). This has been corroborated by in vivo studies, whereby imaging studies may show areas of flow disturbance predisposing to early thrombotic events (Torrealba et al., 2023).

In vitro studies have revealed that CSs provide a more laminar flow than BMSs due to their smoother surfaces and possibly the physical barrier created against arterial wall irregularities (Reijnen, 2020). In vivo studies have proven that CSs are associated with reduced intimal hyperplasia, explaining its lower restenosis rates. Clinical follow‐up studies using CSs have reported that at 36 months, the overall primary patency, assisted primary, secondary patency, and limb salvage rates were 92.2%, 95.7%, 97.8% and 100%, respectively (Shen et al., 2023). Others have suggested that CSs can overcome complex, tortuous anatomy with a thrombus load to improve clinical outcomes. Recent series have confirmed fewer reinterventions for CSs than BMSs and superior patency rates in the short and medium term.

The radial force exerted by a stent on the surrounding arterial wall is a critical factor that influences vascular physiology. In vitro tests have demonstrated that any imbalance in the radial force may lead to stent malposition. This can create zones of turbulent flow, compromising long‐term stent patency (Groot Jebbink, Ter Mors, et al., 2017). In vivo, this has been a concern as mal‐deployed stents have been associated with an increased risk of restenosis and other complications, such as stent fracture or vessel trauma (Piffaretti et al., 2019).

Advancements in KS technology aim to address the physiological challenges posed by traditional stents. There were several challenges with traditional stents, such as inadequate apposition to the wall of vessels, small diameter and excessive length (Kobo et al., 2020). Innovations seek to minimise flow disturbances and improve the haemodynamic profile within the stented segment (Groot Jebbink et al., 2015; Scheinert et al., 1999). In vitro studies support the concept of stents manufactured according to the arterial geometry to provide a physiological flow, reducing the risk of restenosis (Abdelbaqy et al., 2022). Similarly, in vivo research, including clinical trials, has replicated these findings, with recent stent designs demonstrating improved outcomes in blood flow and vessel patency rates (Radosa et al., 2022).

In summary, the impact of KSs on vascular physiology is multifactorial and influenced by both the stent type and design. Combining both in vitro and in vivo evidence contributes to the growing body of knowledge that underscores the importance of considering physiological changes when selecting KSs for endovascular reconstruction. As technology advances, ongoing research into the hemodynamic effects of KSs will continue to inform clinical practice, aiming to optimise outcomes for patients with AIOD. Despite positive results, variability in stent type, deployment techniques and lesion assessment methods across studies complicates direct comparison and generalization. To ensure the long‐term success and applicability of the technique, further research is required (Greiner et al., 2003).

## 4 HOW DOES THE IMPACT ON PHYSIOLOGY ALTER CLINICAL OUTCOMES?

The impact of KSs on vascular physiology is a nuanced aspect of endovascular reconstruction, with significant implications for clinical outcomes. When compared to other methods of aortic bifurcation reconstruction, KSs present unique physiological and clinical ramifications (Groot Jebbink et al., 2015).

Historically, open surgical reconstruction was regarded as the gold standard for AIOD, particularly before the advancement of endovascular techniques. However, what is apparent is the recent trend towards more patients undergoing endovascular techniques. Clinical outcomes following KS intervention depend on maintaining optimal blood flow and minimising the risk of restenosis. Studies have shown that BMSs and CSs restore flow immediately post‐procedure (Moon et al., 2015). However, the long‐term success of KSs can be compromised by the incidence of restenosis, which affects patient morbidity and the need for reintervention (Torrealba et al., 2023).

Alternative methods, such as CERAB, have been developed to address some of the physiological challenges caused by KSs. The CERAB technique, employing CS designed to fit the aortic bifurcation more anatomically, aims to minimise any flow disturbance by reducing turbulence and the associated risk of thrombosis and restenosis (Groot Jebbink et al., 2015; Shen et al., 2023). The CERAB method contrasts the KS approach, where two separate stents are placed in close proximity, which can create areas of turbulent flow at the stent overlap region (Groot Jebbink et al., 2015) (Figure 1).

The CERAB technique has significantly reduced the risk of post‐procedure vessel obstruction and improved blood flow within affected vessels (Reijnen, 2020). This advancement is primarily attributed to the CERAB design, which more closely replicates the natural anatomy of the aortic bifurcation (Taeymans et al., 2016). Comparative studies have highlighted that the mismatch in volume and area associated with traditional kissing stents is notably larger than that observed with CERAB stents. This finding underscores the anatomical and physiological advantages of CERAB in mimicking the human aortic bifurcation (Groot Jebbink et al., 2015). Moreover, traditional kissing stents are frequently linked to flow recirculation zones, which can impair haemodynamic efficiency. In contrast, the CERAB technique minimizes radial mismatch, resulting in significantly smaller zones of recirculation and optimizing haemodynamic performance (Groot Jebbink, Mathai, et al., 2017). This combination of anatomical precision and enhanced haemodynamic properties positions CERAB as a superior intervention for aortic bifurcation reconstruction.

In vivo studies comparing KSs with other reconstruction methods like CERAB or aortoiliac bypass have indicated that whilst KSs are less invasive and offers quicker recovery times, the long‐term patency rates and the need for repeat procedures can be more favourable with procedures such as CERAB (Radosa et al., 2022; Torrealba et al., 2023). Balloon‐expandable stents, another alternative, provide an option for more rigid support in calcified arteries. However, they may not offer the same flexibility as self‐expanding stents used in KS configurations (Soga et al., 2020). While CERAB shows promising early results, much of the supporting evidence is derived from in vitro studies and small, single‐centre series. Long‐term, multi‐centre, randomised trials are lacking, limiting definitive conclusions regarding CERAB's superiority over KSs, particularly in diverse patient populations with varying anatomical complexity.

The selection of an endovascular reconstruction procedure must be carefully considered whilst considering the immediate procedural success with long‐term outcomes. KSs effectively restore blood flow, but alternative methods like CERAB offer advantages in terms of flow dynamics and reduced restenosis rates. The choice of technique should be guided by a thorough assessment of the patient's vascular anatomy. Furthermore, the severity of the arterial disease and the potential for physiological changes post‐intervention should be considered. As endovascular technology evolves, so does the evidence base guiding the appropriate use with the ultimate goal of optimising clinical outcomes for patients with AIOD.

Clinical recommendations to carry forward based on the reviewed literature include:

- TASC A–B lesions: Both BMSs and CSs may be appropriate, with the choice guided by patient comorbidities.
- TASC C–D lesions or severe calcification: CSs preferred due to superior patency and lower complication rates.
- High‐risk surgical patients: KSs with CSs provide a viable alternative to open repair.
- Anatomically complex bifurcations: Consider CERAB for better flow dynamics and reduced restenosis.

## 5 CONCLUSION

Endovascular intervention has become the first line of treatment for AIOD when conservative measures have failed or are not deemed appropriate. Endovascular techniques depend on the patient's arterial anatomy, overall fitness and operator expertise. The deployment of KSs can alter flow dynamics, leading to turbulent flow and increased shear stress. Both these factors may contribute to thrombotic events and subsequent restenosis. Comparative techniques like CERAB are manufactured and used to reduce radial mismatch and create more favourable flow dynamics. This could mean better clinical outcomes compared to traditional KS methods. Both in vitro and in vivo studies have demonstrated the necessity for a strategic approach to stent selection that considers individual patient anatomy and disease severity. The ultimate choice between KSs and other reconstruction techniques must be based principally on the individual clinical symptoms and signs. Further guidance can be based on the length of any arterial stenosis/occlusion and the tortuosity or calibre of the vessel. Lastly, a comprehensive understanding of the potential physiological changes and their impact on clinical outcomes should be incorporated into the decision‐making.

## AUTHOR CONTRIBUTIONS

All authors confirm that they have read and approved the final version of the manuscript and agree to be accountable for all aspects of the work to ensure that questions related to the accuracy or integrity of any part of the work are appropriately investigated and resolved. We also confirm that all persons designated as authors qualify for authorship, and all those who qualify are listed.

## CONFLICT OF INTEREST

D.M.B. is Editor‐in‐Chief of *Experimental Physiology*, Chair of the Life Sciences Working Group, member of the Human Spaceflight and Exploration Science Advisory Committee to the European Space Agency and member of the Space Exploration Advisory Committee to the UK and Swedish National Space Agencies. D.M.B. is also affiliated to Bexorg, Inc. (USA) focused on the technological development of novel biomarkers of cerebral bioenergetic function and structural damage in humans.

## FUNDING INFORMATION

None.

## Data Availability

The evidence used to support this review is publicly available in electronic databases such as PubMed, Ovid, Scopus and Embase.

## References

[eph70020-bib-0001] Abdelbaqy, O. M. , Holewijn, S. , Zeebregts, C. J. , & Reijnen, M. M. (2022). The covered endovascular reconstruction of the aortic bifurcation (CERAB) technique for aorto‐iliac occlusive disease. Surgical Technology International, 40, 263–270. 10.52198/22.STI.40.CV1542 35179733

[eph70020-bib-0002] Ahmad, F. A. , Hennessy, M. M. , & Nath, A. F. (2022). Fate of asymptomatic limb after kissing stents in aortoiliac occlusive disease. Vascular Specialist International, 38, 7. 10.5758/vsi.210074 35361742 PMC8971782

[eph70020-bib-0004] Björses, K. , Ivancev, K. , Riva, L. , Manjer, J. , Uher, P. , & Resch, T. (2008). Kissing stents in the aortic bifurcation—A valid reconstruction for aorto‐iliac occlusive disease. European Journal of Vascular and Endovascular Surgery, 36(4), 424–431. 18692412 10.1016/j.ejvs.2008.06.027

[eph70020-bib-0005] Boulitrop, C. , Jayet, J. , Duprey, A. , Pellenc, Q. , Roussel, A. , Cerceau, P. , Ben Abdallah, I. , & Castier, Y. (2020). From the aortic bifurcation to the groin: long‐term outcomes of covered kissing stent placement in combination with iliofemoral reconstruction for extensive iliofemoral occlusive disease. Annals of Vascular Surgery, 64, 11–16. 10.1016/j.avsg.2019.12.020 31972223

[eph70020-bib-0006] Buccheri, D. , Piraino, D. , Andolina, G. , & Cortese, B. (2016). Understanding and managing in‐stent restenosis: A review of clinical data, from pathogenesis to treatment. Journal of Thoracic Disease, 8(10), E1150–E1162. 27867580 10.21037/jtd.2016.10.93 PMC5107494

[eph70020-bib-0006a] Clair, D. G. , & Beach, J. M. (2015). Strategies for managing aortoiliac occlusions: Access, treatment and outcomes. Expert review of cardiovascular therapy, 13(5), 551–563. 10.1586/14779072.2015.1036741 25907618 PMC4717320

[eph70020-bib-0006b] García, L. F. , Gómez‐Rodríguez, J. C. , Cabrera‐Vargas, L. F. , Contreras, M. , Lozada‐Martínez, I. D. , & Rahman, S. (2021). Midterm outcomes of the covered endovascular reconstruction of the aortic bifurcation for aortoiliac occlusive disease in a latinoamerican population. International journal of surgery case reports, 88, 106572. 10.1016/j.ijscr.2021.106572 34749174 PMC8578036

[eph70020-bib-0006c] Greiner, A. , Mühlthaler, H. , Neuhauser, B. , Waldenberger, P. , Dessl, A. , Schocke, M. F. , Jaschke, W. , & Fraedrich, G. (2005). Does stent overlap influence the patency rate of aortoiliac kissing stents?. Journal of endovascular therapy: An official journal of the International Society of Endovascular Specialists, 12(6), 696–703. 10.1583/06-1633.1 16363899

[eph70020-bib-0008] Greiner, A. , Dessl, A. , Klein‐Weigel, P. , Neuhauser, B. , Perkmann, R. , Waldenberger, P. , Jaschke, W. , & Fraedrich, G. (2003). Kissing stents for treatment of complex aortoiliac disease. European Journal of Vascular and Endovascular Surgery, 26(2), 161–165. 12917831 10.1053/ejvs.2002.1882

[eph70020-bib-0009] Groot Jebbink, E. , Grimme, F. A. B. , Goverde, P. C. J. M. , van Oostayen, J. A. , Slump, C. H. , & Reijnen, M. M. P. J. (2015). Geometrical consequences of kissing stents and the covered endovascular reconstruction of the aortic bifurcation configuration in an in vitro model for endovascular reconstruction of aortic bifurcation. Journal of Vascular Surgery, 61(5), 1306–1311. 24486037 10.1016/j.jvs.2013.12.026

[eph70020-bib-0010] Groot Jebbink, E. , Holewijn, S. , Slump, C. H. , Lardenoije, J.‐W. , & Reijnen, M. M. P. J. (2017). Systematic review of results of kissing stents in the treatment of aortoiliac occlusive disease. Annals of Vascular Surgery, 42, 328–336. 10.1016/j.avsg.2017.01.009 28390920

[eph70020-bib-0011] Groot Jebbink, E. , Mathai, V. , Boersen, J. T. , Sun, C. , Slump, C. H. , Goverde, P. C. J. M. , Versluis, M. , & Reijnen, M. M. P. J. (2017). Hemodynamic comparison of stent configurations used for aortoiliac occlusive disease. Journal of Vascular Surgery, 66(1), 251–260.e1. 27743806 10.1016/j.jvs.2016.07.128

[eph70020-bib-0012] Groot Jebbink, E. , Ter Mors, T. G. , Slump, C. H. , Geelkerken, R. H. , Holewijn, S. , & Reijnen, M. M. (2017). In vivo geometry of the kissing stent and covered endovascular reconstruction of the aortic bifurcation configurations in aortoiliac occlusive disease. Vascular, 25(6), 635–641. 28530484 10.1177/1708538117708912 PMC5714162

[eph70020-bib-0013] Hajibandeh, S. , Hajibandeh, S. , Antoniou, S. A. , Torella, F. , & Antoniou, G. A. (2016). Covered vs uncovered stents for aortoiliac and femoropopliteal arterial disease: A systematic review and meta‐analysis. Journal of Endovascular Therapy, 23(3), 442–452. 27099281 10.1177/1526602816643834

[eph70020-bib-0014] Hardman, R. L. , Jazaeri, O. , Yi, J. , Smith, M. , & Gupta, R. (2014). Overview of classification systems in peripheral artery disease. Seminars in Interventional Radiology, 31(4), 378–388. 25435665 10.1055/s-0034-1393976 PMC4232437

[eph70020-bib-0015] Hinnen, J. W. , Konickx, M. A. , Meerwaldt, R. , Kolkert, J. L. P. , van der Palen, J. , Huisman, A. B. , & Geelkerken, R. H. (2015). Long term results of kissing stents in the aortic bifurcation. Acta Chirurgica Belgica, 115(3), 191–197. 26158249 10.1080/00015458.2015.11681095

[eph70020-bib-0015a] Kimyaghalam, A. , Fitzpatrick, N. J. , & Khan, Y. S. (2024). Aortoiliac Occlusive Disease. In StatPearls. StatPearls Publishing. 32644512

[eph70020-bib-0016] Kim, H. O. , Yim, N. Y. , Kim, J. K. , Kang, Y. J. , & Lee, B. C. (2019). Endovascular aneurysm repair for abdominal aortic aneurysm: A comprehensive review. Korean Journal of Radiology, 20(8), 1247. 31339013 10.3348/kjr.2018.0927 PMC6658877

[eph70020-bib-0017] Kobo, O. , Saada, M. , Meisel, S. R. , Hellou, E. , Frimerman, A. , Fanne, R. A. , Mohsen, J. , Danon, A. , & Roguin, A. (2020). Modern stents: Where are we going? Rambam Maimonides Medical Journal, 11(2), e0017. 32374258 10.5041/RMMJ.10403 PMC7202450

[eph70020-bib-0018] Lee, C. W. , Huh, U. , Bae, M. , Han, C. , Kwon, H. , & Kim, G. (2023). Comparison between kissing stents and direct surgical bypass for aortoiliac occlusive disease. Journal of Chest Surgery, 56(4), 264–271. 37096251 10.5090/jcs.23.012 PMC10345658

[eph70020-bib-0019] Mayor, J. , Branco, B. C. , Chung, J. , Montero‐Baker, M. F. , Kougias, P. , Mills, J. L. , & Gilani, R. (2019). Outcome comparison between open and endovascular management of TASC II D aortoiliac occlusive disease. Annals of Vascular Surgery, 61, 65–71.e3. 10.1016/j.avsg.2019.06.005 31394230

[eph70020-bib-0020] Mohamed, F. , Sarkar, B. , Timmons, G. , Mudawi, A. , Ashour, H. , & Uberoi, R. (2002). Outcome of ‘kissing stents’ for aortoiliac atherosclerotic disease, including the effect on the non‐diseased contralateral iliac limb. Cardiovascular and Interventional Radiology, 25(6), 472–475. 12357313 10.1007/s00270-001-0120-9

[eph70020-bib-0021] Moon, J. Y. , Hwang, H. P. , Kwak, H. S. , Han, Y. M. , & Yu, H. C. (2015). The results of self‐expandable kissing stents in aortic bifurcation. Vascular Specialist International, 31(1), 15–19. 26217639 10.5758/vsi.2015.31.1.15 PMC4480290

[eph70020-bib-0022] Mwipatayi, B. P. , Sharma, S. , Daneshmand, A. , Thomas, S. D. , Vijayan, V. , Altaf, N. , Garbowski, M. , & Jackson, M. , & COBEST co‐investigators . (2016). Durability of the balloon‐expandable covered versus bare‐metal stents in the covered versus balloon expandable stent trial (COBEST) for the treatment of aortoiliac occlusive disease. Journal of Vascular Surgery, 64(1), 83–94.e1. 27131926 10.1016/j.jvs.2016.02.064

[eph70020-bib-0023] Mwipatayi, B. P. , Thomas, S. , Wong, J. , Temple, S. E. L. , Vijayan, V. , Jackson, M. , & Burrows, S. A. , & Covered Versus Balloon Expandable Stent Trial (COBEST) Co‐investigators . (2011). A comparison of covered vs bare expandable stents for the treatment of aortoiliac occlusive disease. Journal of Vascular Surgery, 54(6), 1561–1570.e1. 21906903 10.1016/j.jvs.2011.06.097

[eph70020-bib-0024] Nanto, K. , Iida, O. , Fujihara, M. , Yokoi, Y. , Tomoi, Y. , Soga, Y. , Fujita, M. , Masuda, M. , Okamoto, S. , Ishihara, T. , Kanda, T. , Tsujimura, T. , Matsuda, Y. , Okuno, S. , & Mano, T. (2019). Five‐year patency and its predictors after endovascular therapy for aortoiliac occlusive disease. Journal of Atherosclerosis and Thrombosis, 26(11), 989–996. 30996200 10.5551/jat.45617 PMC6845694

[eph70020-bib-0025] Piffaretti, G. , Fargion, A. T. , Dorigo, W. , Pulli, R. , Gattuso, A. , Bush, R. L. , Pratesi, C. , Fontana, F. , Piacentino, F. , Castelli, P. , Speziali, S. , Angiletta, D. , Marinazzo, D. , Zacà, S. , Grego, F. , Antonello, M. , Piazza, M. , Squizzato, F. , Bellosta, R. , … & Turchino, D. (2019). Outcomes from the multicenter italian registry on primary endovascular treatment of aortoiliac occlusive disease. Journal of Endovascular Therapy, 26(5), 623–632. 31331235 10.1177/1526602819863081

[eph70020-bib-0026] Radosa, C. G. , Reeps, C. , Nebelung, H. , Schön, F. , & Hoffmann, R. T. (2022). Covered endovascular reconstruction of aortic bifurcation (CERAB). Radiologie, 62(7), 601–606. 35352137 10.1007/s00117-022-00989-6

[eph70020-bib-0027] Reijnen, M. M. (2020). Update on covered endovascular reconstruction of the aortic bifurcation. Vascular, 28(3), 225–232. 31896301 10.1177/1708538119896197

[eph70020-bib-0028] Scheinert, D. , Schröder, M. , Balzer, J. O. , Steinkamp, H. , & Biamino, G. (1999). Stent‐supported reconstruction of the aortoiliac bifurcation with the kissing balloon technique. Circulation, 100(suppl_2), II–295. 10567319 10.1161/01.cir.100.suppl_2.ii-295

[eph70020-bib-0029] Serefli, D. , Saydam, O. , Engin, A. Y. , & Atay, M. (2021). Midterm results of kissing stent reconstruction of the aortoiliac bifurcation. Annals of Surgical Treatment and Research, 101(4), 247. 34692597 10.4174/astr.2021.101.4.247 PMC8506018

[eph70020-bib-0030] Sharafuddin, M. J. , Hoballah, J. J. , Kresowik, T. F. , Sharp, W. J. , Golzarian, J. , Sun, S. , & Corson, J. D. (2008). Long‐term outcome following stent reconstruction of the aortic bifurcation and the role of geometric determinants. Annals of Vascular Surgery, 22(3), 346–357. 18411026 10.1016/j.avsg.2007.12.013

[eph70020-bib-0031] Shen, C.‐Y. , Qu, C.‐J. , Zhang, Y.‐B. , Fang, J. , Teng, L.‐Q. , & Li, J.‐L. (2023). Midterm outcomes of kissing covered self‐expanding stents for reconstruction of complex aortoiliac occlusive disease. Annals of Vascular Surgery, 94, 239–245. 10.1016/j.avsg.2023.02.011 36870565

[eph70020-bib-0032] Soga, Y. , Nakata, M. , & Ando, K. (2020). Treatment for aortoiliac bifurcation disease by balloon‐expandable covered stent; “Double‐D” molding technique. Journal of Cardiology Cases, 22(3), 143–146. 32884599 10.1016/j.jccase.2020.05.024 PMC7452355

[eph70020-bib-0033] Squizzato, F. , Piazza, M. , Pulli, R. , Fargion, A. , Piffaretti, G. , Pratesi, C. , Grego, F. , & Antonello, M. , & ILIACS Registry Group . (2021). Covered versus bare metal kissing stents for reconstruction of the aortic bifurcation in the ILIACS registry. Journal of Vascular Surgery, 73(6), 1980–1990.e4. 33253875 10.1016/j.jvs.2020.10.066

[eph70020-bib-0034] Taeymans, K. , Goverde, P. , Lauwers, K. , & Verbruggen, P. (2016). The CERAB technique: Tips, tricks and results. The Journal of Cardiovascular Surgery, 57(3), 343–349. 27012930

[eph70020-bib-0035] Torrealba, J. I. , Blessing, E. , Rohlffs, F. , Panuccio, G. , Carpenter, S. , & Kölbel, T. (2023). Single access covered endovascular reconstruction of the aortic bifurcation. Journal of Vascular Surgery Cases, Innovations and Techniques, 9(4), 101343. 10.1016/j.jvscit.2023.101343 PMC10641678 37965110

[eph70020-bib-0035a] Vértes, M. , Juhász, I. Z. , Nguyen, T. D. , Veres, D. S. , Hüttl, A. , Nemes, B. , Hüttl, K. , & Dósa, E. (2018). Stent Protrusion >20 mm Into the Aorta: A New Predictor for Restenosis After Kissing Stent Reconstruction of the Aortoiliac Bifurcation. Journal of endovascular therapy: An official journal of the International Society of Endovascular Specialists, 25(5), 632–639. 10.1177/1526602818794959 30122138

[eph70020-bib-0035b] Yang, M. , Zhang, B. , Niu, G. , Yan, Z. , Tong, X. , & Zou, Y. (2021). Long‐term results of endovascular reconstruction for aortoiliac occlusive disease. Quantitative imaging in medicine and surgery, 11(4), 1303–1312. 10.21037/qims-20-599 33816169 PMC7930674

[eph70020-bib-0036] Zeng, C. , Wu, Z. , Lei, J. , Pu, H. , Qiu, P. , Peng, Z. , Liu, Y. , Ye, K. , & Lu, X. (2023). Covered stents vs bare metal stents for aortoiliac arterial diseases: A systematic review and meta‐analysis. Journal of Endovascular Therapy, 15266028231212761. 10.1177/15266028231212761 38031669

